# Non-destructive monitoring method for leaf area of *Brassica napus* based on image processing and deep learning

**DOI:** 10.3389/fpls.2023.1163700

**Published:** 2023-07-18

**Authors:** Mengcheng Li, Yitao Liao, Zhifeng Lu, Mai Sun, Hongyu Lai

**Affiliations:** ^1^ College of Engineering, Huazhong Agricultural University, Wuhan, China; ^2^ College of Resources and Environment, Huazhong Agricultural University, Wuhan, China

**Keywords:** leaf area, deep learning, image segmentation, monitor, effect of growth

## Abstract

**Introduction:**

Leaves are important organs for photosynthesis in plants, and the restriction of leaf growth is among the earliest visible effects under abiotic stress such as nutrient deficiency. Rapidly and accurately monitoring plant leaf area is of great importance in understanding plant growth status in modern agricultural production.

**Method:**

In this paper, an image processing-based non-destructive monitoring device that includes an image acquisition device and image process deep learning net for acquiring *Brassica napus* (rapeseed) leaf area is proposed. A total of 1,080 rapeseed leaf image areas from five nutrient amendment treatments were continuously collected using the automatic leaf acquisition device and the commonly used area measurement methods (manual and stretching methods).

**Results:**

The average error rate of the manual method is 12.12%, the average error rate of the stretching method is 5.63%, and the average error rate of the splint method is 0.65%. The accuracy of the automatic leaf acquisition device was improved by 11.47% and 4.98% compared with the manual and stretching methods, respectively, and had the advantages of speed and automation. Experiments on the effects of the manual method, stretching method, and splinting method on the growth of rapeseed are conducted, and the growth rate of rapeseed leaves under the stretching method treatment is considerably greater than that of the normal treatment rapeseed.

**Discussion:**

The growth rate of leaves under the splinting method treatment was less than that of the normal rapeseed treatment. The mean intersection over union (mIoU) of the UNet-Attention model reached 90%, and the splint method had higher prediction accuracy with little influence on rapeseed.

## 1 Introduction

Leaves are very important organs in plants and are often monitored to reflect plant growth. In the late last century, leaf monitoring was mainly performed by manual measurement (Ross et al., 2000). With the development of computer technology, image processing is increasingly applied in the field of plant growth monitoring (Hamuda et al., 2016). Therefore, the accurate acquisition of leaf image information has become the focus of plant growth monitoring. Additionally, non-destructive continuous monitoring has become a hot topic in recent years. In particular, plant-specific tracking observation techniques are key to obtaining morphological parameters of plant dynamics over time (Ali et al., 2019; Su et al., 2020; Mohd Asaari et al., 2022). Monitoring crop growth is important for precision agriculture, allowing scientific and efficient cultivation to improve crop yields and reduce the waste of resources. Leaf area (LA) reflects the performance of mechanisms such as radiation shielding, water and energy exchange, plant growth, and biological productivity (Hsiao et al., 1970; Salah and Tardieu, 1997). Accurate continuous measurement of LA helps in understanding leaf ontogenesis, especially under multiple plant–environment interactions for researchers (Mielewczik et al., 2013; Friedli and Walter, 2015). Plants often encounter nutrient-improvised conditions, retarding leaf growth, decreasing LA, and reducing photosynthetic capacity. The reduction in LA usually arises first when plants are exposed to nutrient deficiency (Lu et al., 2016b). A reasonable estimation of LA and its variation would be useful to elucidate plant response mechanisms or phenotypes to nutrients or the environment, hence guiding the subsequent cultivation management.

The current methods for monitoring leaf growth are mainly manual destructive measurements and non-destructive monitoring based on computer vision. Traditional manual plant monitoring methods rely on the professional measuring experience of the people involved, and sophisticated instruments are used to measure various morphological parameters of the leaves (Jonckheere et al., 2004). It costs much time and money to train professionals with the ability to monitor plants, even well-trained workers will make mistakes in monitoring the plants’ status, and there are few institutions dedicated to providing this type of training. In addition, the manual measurement of the measurement angle, leaf attitude, and other factors also affects the accuracy of the measurement. Another difficulty is the time required to manually assess the growth status of plants, which hinders rapid decision-making and large-scale assessments. Manual measurement often causes minimal damage to the leaf.

The continuous development of image processing technology in recent years and the appearance of many inexpensive image processing devices in the market allow the use of image processing technology in agriculture at a more extensive and easier stage. Non-destructive measurements based on computer vision include an online monitoring platform for plants based on 3D stereo reconstruction technology and a 2D image acquisition platform based on industrial cameras.

Plant monitoring methods based on 3D reconstruction techniques are used in many plant morphology studies. Apelt et al. (2015) built a 3D reconstruction platform using a light field camera to reconstruct the *Arabidopsis thaliana* wild-type in 3D and analyzed the information of individual leaf morphology and the time of leaf appearance in time series, providing a new idea for accurate plant morphology measurement and growth-related characteristics. Cuevas Velasquez et al. (2020) used a pair of stereo images obtained by the camera to reconstruct the rose in three dimensions, calculating the skeleton and branching structure as a basis for later pruning using the gardening robot. Although 3D reconstruction can reflect the overall morphology of the plant with high accuracy, there are problems, such as low efficiency and high cost for leaf measurement, due to expensive equipment and high setup and maintenance costs. Intensive monitoring in a very short amount of time is not possible because it takes considerable time to complete 3D reconstruction. Deep learning is a powerful tool for building 3D image processing models, but thus far, there are still problems such as too few samples and difficulties in annotating datasets when applying deep learning to 3D model reconstruction (Li et al., 2020).

Obtaining information from two-dimensional plant images through image processing algorithms is an efficient measurement method that uses digital images and time series to acquire plant phenotypic data using various methods and sensors in controlled environments and the field. There are precedents for using two-dimensional image processing techniques for LA, plant growth status, disease diagnosis, plant vigor, and postharvest vegetable quality monitoring (Pipatsitee et al., 2019; Baar et al., 2022). One of those methods is to use cameras to collect images and then import them into computer-side or smartphone-side software for batch processing, which further improves the accuracy of measurement and processing speed. Measurements are taken with a camera or smartphone, and the images are placed in software such as MATLAB or Image-Pro Plus to calculate the leaf area with the aid of a computer (Lu et al., 2016a; Badiger et al., 2022). Julian Schrader presented Leaf-IT, an application for measuring leaf area and other trait-related areas on smartphones (Schrader et al., 2017). Although this method can obtain high-precision leaf area data, it is destructive to the plant and cannot obtain continuously changing data of leaf area over time on the same plant. Another method is to fix light black beads around the leaf, use thin wire fixation to spread the leaf, and use a camera to photograph the leaf vertically (Mielewczik et al., 2013; Friedli and Walter, 2015). The stretching method mentioned above is usually used to measure the leaf area of soybean; in rapeseed, the results may be inaccurate. The force pull effect on leaf growth could not be assessed. Plant growth needs to be monitored as much as possible without affecting the natural growth state of the plant and at a high degree of automation. Therefore, there is some room for improvement in the stretching method in terms of the effect on the leaf.

In this study, a plywood-based leaf area acquisition device was designed to obtain leaf area based on a deep learning-based image semantic segmentation technique. The device adopts the form of clamping the whole leaf flat, reducing the influence of errors caused by leaf folds. Compared to the tensile method, the mechanical damage to the leaf is reduced. We use different deep learning models for the semantic segmentation of leaves and propose a U-Net model that incorporates attention block, which has higher accuracy than other models. The accuracy of the splint measuring method is compared with that of the manual method and the stretching method. The effects of various methods on the growth of rapeseed were also investigated. The results showed that the splint method proposed in this paper had an acquisition error of leaf area within ±5%, which provides a feasible approach for non-destructive measurement of the rapeseed leaf area.

## 2 Materials and methods

### 2.1 Hardware setup

**Figure 1:**
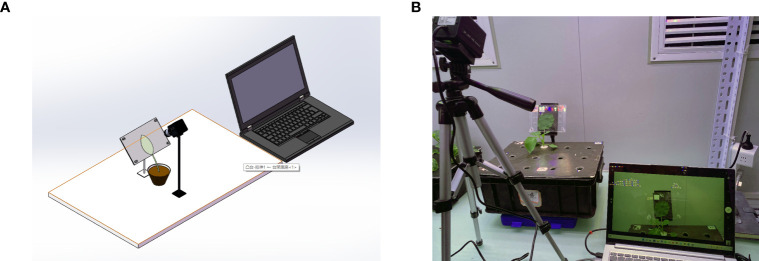
**(A)** 3D view of the device. **(B)** Image acquisition device. This device consists of an industrial camera, a splint, and a laptop. The computer drives the industrial camera, and the image of the blade is captured by the image sensor in the camera and transferred to the laptop for storage through the USB connection cable.

A complementary metal oxide semiconductor (CMOS) camera was used to obtain the leaf images and pass them into the leaf segmentation model located in the computer (Computer with AMD Ryzen 5 3500X processor, memory 16G, operating system Microsoft Windows 10 Professional, compiler Pycharm) for segmentation. Based on the switching power supply, LM2596 DC step-down module, electromagnetic relay and STM32 microcontroller, and other equipment, a set of night light control systems was built. The system is shown in **Figure 1**. The PC side and the microcontroller used serial communication, and the microcontroller obtained the light command, controlled the relay on and off to complete the light command response, and achieved an automatic night light switch.

### 2.2 Plant material

Rapeseed seedlings were grown in an environmentally controlled growth chamber located in the College of Resources and Environment, Huazhong Agricultural University, with a 14-h photoperiod under a photosynthetic photon flux density (PPFD) of 250 μmol m^−2^ s^−1^ at the leaf level. The temperature was 20°C during the day and 18°C at night, and the humidity in the greenhouse was controlled between 50% and 60%. Seeds were germinated for 7 days on gauze floating on the surface of deionized water in a dish. First, 80 uniform seedlings were transplanted into half-strength nutrient solution in 8.0-L black plastic containers. Seedlings were grown under a half-strength nutrient solution for 6 days before they were transplanted to a full-strength nutrient solution. The 13-day-old seedlings were transplanted to the full-strength nutrient solution, comprising, 3 mM of NH_4_NO_3_, 0.28 mM of Na_2_HPO_4_, 0.641 mM of NAH_2_PO_4_, 2 mM of KCl, 3.24 mM of CaCl_2_, 2 mM of MgCl_2_, 1 mM of Na_2_SO_4_, 4.6 μM of H_2_BO_3_, 9 μM of MnCl_2_, 0.3 μM of CuSO_4_, 0.8 μM of ZnSO_4_, 0.1 μM of Na_2_MoO_4_, 0.1 μM of Na_2_MoO_4_·2H_2_O, 0.1 μM of H_32_Mo_7_N_6_O_28_, 0.05 mM of FeSO_4_, and 0.05 mM of Na2EDTA. Five different treatments (CK\-N\-P\-K\-Mg) were used: control (CK, full nutrient), -N (0 mM of NH_4_NO_3_), -P (0 mM of Na_2_HPO_4_ and NAH_2_PO_4_), -K (0 mM of KCl), and -Mg (0 mM of MgCl_2_). The nutrient solution was renewed every 3 days. During hydroponic cultivation, the nutrient solution was aerated for 0.5 h with an air pump that bubbled air through air stones every 2 h to maintain a water oxygen content of approximately 8.0 mg/L.

### 2.3 Measuring methods

Three images were collected for each plant by selecting a leaf, and the daytime and night-time acquisitions are listed in **Table 1**. In the stretching method, leaves were stretched by five small clips with a proper counterweight, and images were captured by a camera installed directly above the leaves.

**Table 1:** Acquisition of image datasets.

Day/night group	Method			
	Manual	Splint	Stretching	Total
-CK	36	36	36	108
-N	36	36	36	108
-P	36	36	36	108
-K	36	36	36	108
-MG	36	36	36	108
Total	180	180	180	540

#### 2.3.1 Manual method and stretching method

**Figure 2:**
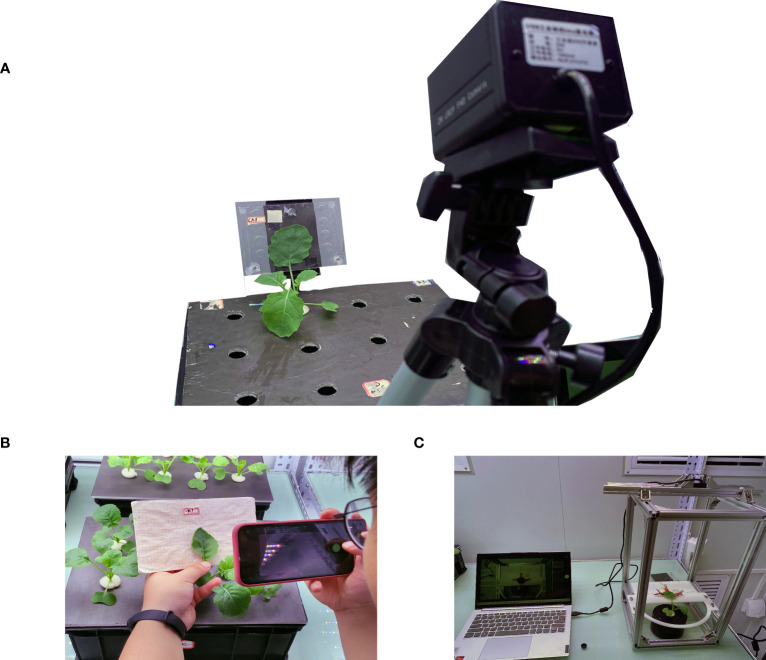
**(A)** Splint method. **(B)** Manual method. **(C)** Stretching method.


In the manual method, the front view image of the rapeseed leaf was taken by a mobile phone and put into computer software for analysis shown in **Figure 2B**. First, the preprepared calibration board was placed behind the rapeseed leaves to be tested, and a smartphone was used to take a frontal image of the rapeseed, which was stored in the phone’s built-in memory. After photos were taken, the images in the phone were exported to computer software, IPP6.0 (Image-pro Plus 4.5 software) (Media Cybernetics, Silver Spring, MD, USA) to process the image. In the software, first, the leaf area was manually selected, the number of pixels $PNL_{C}$ (pixel number of a leaf) was written down, and then the area of the known area $S_{C}$ (5 mm × 5 mm) was selected. The number of pixels is denoted as $PNC_{C}$ (pixel number of calibration objects) and then the leaf area as $LA_{C}$ :

(1) $$LA_{c}=\frac{PNL_{c}\times S_{c}}{PNC_{c}}.$$

$LA_{C}$ is the leaf area, $PNL_{C}$ is the pixel number of leaf, and $PNC_{C}$ is the pixel number of calibration objects.

In the stretching method which is shown in **Figure 2C**, each leaf was fixed to the camera’s focusing plane, supported by a plate with a white background, and evenly clamped to the five points of the leaf by five small clips connected by thin lines with a counterweight consisting of a centrifugal tube filled with an appropriate amount of water, hanging around the edge of the ring. The camera was fixed 30 cm above the leaf, and the lens was parallel to the leaf. A set of computer-controlled lighting systems was set up on the bench to enable the night camera to work properly. Before an image was obtained, the calibration board was fixed on the background plate, a 5 mm × 5 mm area on the calibration board was taken as the calibration area ( $S_{St}$ ), and the number of pixels $PNS_{St}$ (pixel number of stretching) were recorded. The actual area of the marked area is $\;S_{St}$ . The number of pixels in the leaf area calculated in the algorithm was taken as $PNL_{St}$ (pixel number of stretching methodPixel number of Stretching Method) and then the leaf area as $LA_{St}$ :


(2) $$LA_{St}=\frac{PNL_{St}\times S_{St}}{PNC_{St}}.$$

$LA_{St}$ is the leaf area of the stretching method, and $PNL_{St}$ is the pixel number of the leaf area in the stretching method.

#### 2.3.2 Splint method

In this method, the monitored leaf was fixed to the camera imaging plane by two transparent acrylic plates, which were fixed to the greenhouse stand by a rubber base, and the two acrylic plates were connected by bolts and nuts it is shown in **Figure 2A**. Additionally, the plywood lamination gap can be adjusted by rotating the bolts to suit different thicknesses of the leaf. During the image acquisition process, the light was fixed by a variable bracket approximately 50 cm directly above the camera and approximately 100 cm away from the object. The device was set to leave the light on during the day, turn on the light 1 s before photographing the image at night, and turn off the light 1 s after images were taken. Before image capture, the calibration board was fixed on the acrylic plate, a picture was taken as the calibration image, and the 5 mm × 5 mm size area was employed on the calibration board as the calibration area ( $S_{Sp}$ ). We note down the number of pixel points was taken as $PN_{Sp}$ (pixel number of the splint method) and the number of pixel points as $PNL_{Sp}$ (pixel number of the leaf area) as calculated in the algorithm. Then, the leaf area was calculated as follows:


(3) $$LA_{Sp}=\frac{PNL_{Sp}\times S_{Sp}}{PNC_{Sp}}.$$

$LA_{Sp}$ is the leaf area of the splint method, and $PNL_{Sp}$ is the pixel number of the leaf area in the splint method.

#### 2.3.3 Calibration leaf area

To compare the effectiveness of the three measurement methods, the measured leaves were cut out and placed into the scanner for scanning, and leaf area S was measured using the leaf analysis software as the standard value. The measurement accuracy (Acc) is expressed as follows:


(4) $$Acc=\frac{\left|S-S_{t}\right|}{S}\times 100\%.$$

### 2.4 Image segmentation model

Image annotation is an important step in the extraction of rapeseed leaves using deep learning methods. The semantic segmentation of the image is compiled by using the Labelme software, which separates the rapeseed leaves from the image background and obtains the json format file with the boundary points of the leaves, and the masked image of the rapeseed leaves is obtained after the segmentation process. The rapeseed leaf segmentation dataset was randomly divided into a training set, test set, and validation set with a ratio of 6:2:2. The training set, test set, and validation set contain 648, 216, and 216 images, respectively, for a total of 1,080 images.

#### 2.4.1 PSPNet

**Figure 3:**
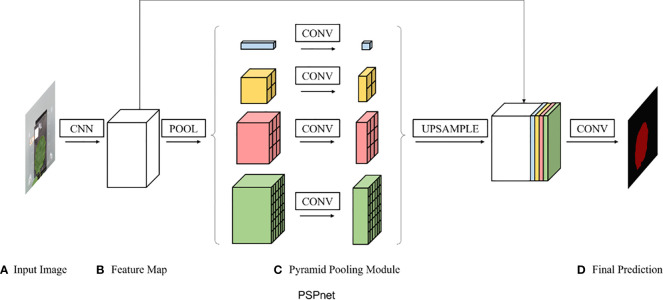
PSPNet structure.

PSPNet was proposed in 2017 by Zhao et al. (2017) and was improved based on fully convolutional network (FCN). The backbone part uses ResNet50 as the feature extraction model, and the feature layers extracted from the backbone part are fused under four different scales using a pyramid module. The pyramid module is actually a four-level module, the top level is the global average pooling, and levels 2, 3, and 4 divide the input feature layers into 2×2, 3×3, and 6×6 subregions, respectively. The pooling was averaged for each subregion separately. Finally, a 3×3 convolution was used for feature integration, and a 1×1 convolution was used for channel adjustment. The PSPNet model structure is shown in **Figure 3**.

#### 2.4.2 DeepLab v3+

**Figure 4:**
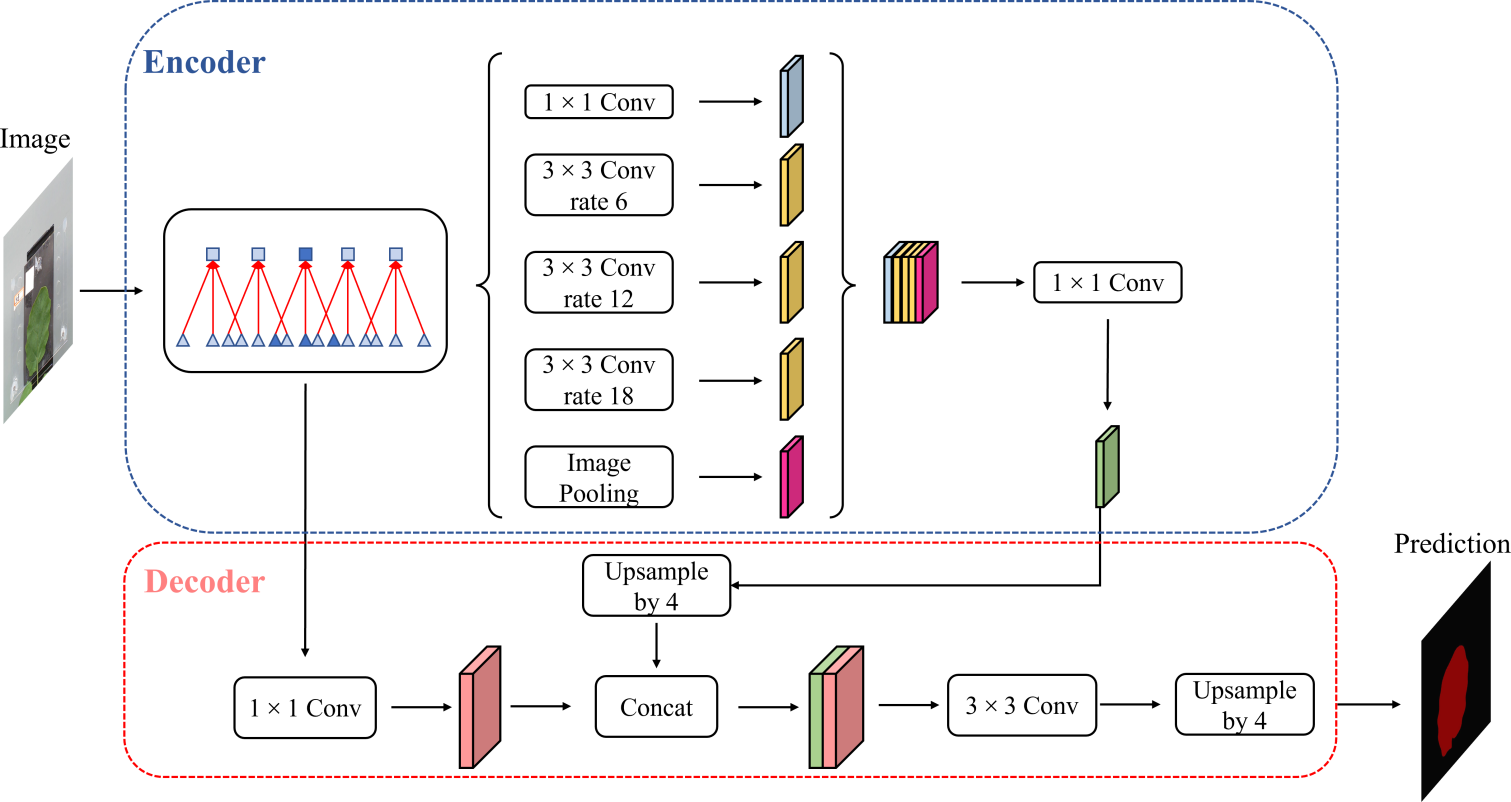
DeepLab V3+ structure.

DeepLab v3+ is a new semantic segmentation algorithm that was introduced by Google in 2018, improving on the DeepLab v1-3. Cai introduced the attention mechanism into the DeepLab v3+ model as shown in **Figure 4**, enhanced the feature information of strawberry images, and improved the segmentation accuracy by adjusting the weights of the feature channels during the propagation of the neural model through the attention mechanism (Cai et al., 2022). Peng et al. applied DeepLab v3+ to the segmentation of litchi branches and used migration learning and data enhancement methods to accelerate model convergence and improve model robustness (Peng et al., 2020). Therefore, the DeepLab v3+ model was selected as a semantic segmentation model method in this study.

#### 2.4.3 U-Net

For the segmentation model, we used U-Net architecture, which is proposed by Ronneberger et al. (2015), a deep learning-based image segmentation model. Deep learning-based semantic segmentation algorithms classify images at the pixel level. The input image is processed by the deep neural model. The pixels in the image are encoded by the convolution layer and pooling layer in the downsampling process of the model and then decoded by deconvolution in the upsampling process. Finally, the segmented image is obtained.

**Figure 5:**
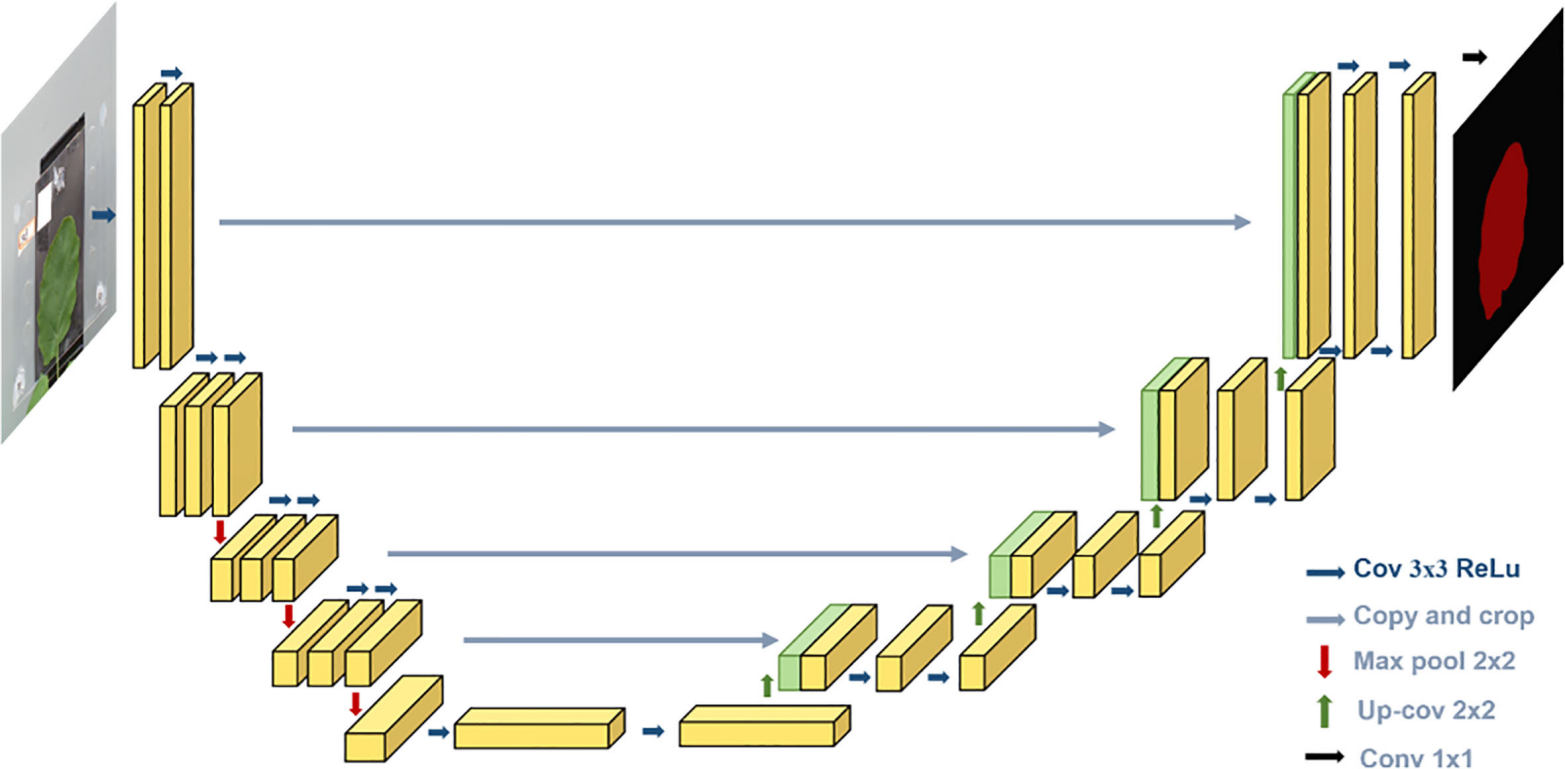
U-Net structure.

As shown in **Figure 5**, the U-Net is a deep learning model with a typical encoding–decoding structure. It has a left–right symmetric structure, similar to the letter, with the encoding part of the model on the left and the decoding part on the right. The advantage of the U-Net model is that it can achieve high segmentation accuracy with relatively less data.

The “encoding” part uses VGG16 as the main feature extraction part, with five layers. Each layer uses two 3 * 3 convolutional kernels, each followed by a rectified linear unit (ReLU) activation function and a 2 * 2 maximum pooling operation. The “decoding” part uses the five effective feature layers obtained from the “encoding” part for feature fusion by upsampling and stacking the feature layers.

The encoding part consists of four submodules, each of which downsamples the model of the previous level by a factor of two, and the resolution of the image decreases to one-half of the original with every module. The decoding part is similar in structure to the encoding part and consists of four submodules, each of which upsamples the input image by a factor of two, and with each passing submodule, the resolution of the image rises to two times that of the input image. The loss function with boundary weights in the loss function is formulated as follows:


(5) $$\text{Dice=}\frac{2\left|A\cap B\right|}{\left|A\right|+\left|B\right|},$$

where *A* is the *a priori* mask and *B* is the predicted mask.

#### 2.4.4 Attention block

**Figure 6:**
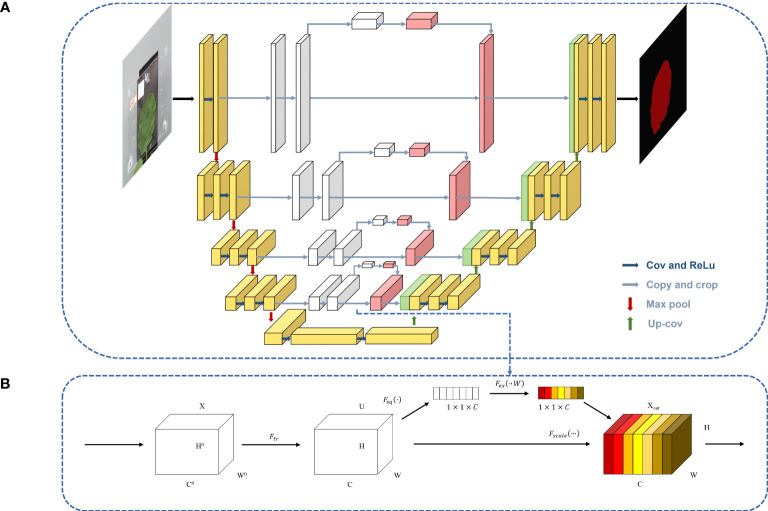
The U-Net-Attention model.

The attention model was first introduced in the seq2seq model (Sutskever, et al., 2014). It is now widely used in different types of machine learning tasks such as natural language processing and image processing as well as speech recognition. According to the different domains where the attention mechanism is applied, the attention weights are applied in different ways and locations, and the attention mechanism is divided into three kinds: spatial domain, channel domain, and hybrid domain. Among them, channel attention has a strong generalization in the image processing domain, so this paper adds channel attention to the channel domain. Channel attention is similar to applying a weight to each channel’s feature map, which represents the relevance of that channel to the key information, and the larger the weight, the higher the relevance. By adding the SE attention block in the fusion stage of the shallow features and deep features in the U-Net model, the shallow features generated in the downsampling procedure are processed by the channel attention and then fused with the subsequent features to achieve the goal of improving the prediction accuracy, and the U-Net-attention structure is shown in **Figure 6A**.

After the feature map is passed in, it is changed into a C-channel feature map with the length and width of H and W, respectively, by one convolution and then into a 3D matrix of size 1◊1◊C by one global average pooling. Then, the weights of each channel are fixed to between 0 and 1 by two full joins and one sigmoid, at which time the matrix corresponds to the weights of each channel in the input layer, and finally, these weights are multiplied by the original input feature layer, which completes channel attention processing. The SE block is shown in **Figure 6B**.

### 2.5 Evaluation of the model

In this paper, we use mean intersection over union (mIoU), mPA, mPrecision, and recall, which are usually used for segmentation tasks as the evaluation metrics, and each metric is calculated as follows:


(6) $$\text{MIoU=}\frac{\text{TP}}{\text{FN+TP+FP}},$$

(7) $$\text{Accuracy=}\frac{\text{TP+TN}}{\text{TP+TN+FP+FN}}\times 100\%,$$

(8) $$\text{Recall=}\frac{\text{TP}}{\text{TP+TN}}\times 100\%,$$

(9) $$\text{Precision=}\frac{\text{TP}}{\text{TP+FP}}\times 100\%.$$

TP , FP , FP , and FN  are from the confusion matrix. The confusion matrix is widely used in the field of machine learning, it is also known as a likelihood matrix or error matrix, and it is a visualization tool.

### 2.6 Effect of the measuring method on the growth experiment

To investigate the effect of continuous monitoring on rapeseed leaves, we also conducted a 7-day continuous monitoring experiment in which leaf area information was collected daily, and the change in the area for seven consecutive days was used as an indicator to evaluate the effect of the monitoring device on rapeseed growth.

We selected the same batch of rape seeds for cultivation, from which 12 seedlings with similar growth were selected and transferred into specific cultivation containers when they were 8 days old. When the seedlings reached 14 days old, they were treated in batches and divided into blank, stretch, and splint treatments, with the following nutrient solution ratios.

Monitoring started at the seedling age of 24 days and continued for eight consecutive days. The leaf area was measured every morning at 08:00. The measurement method was the manual method.

## 3 Results

### 3.1 Accuracy of different measuring methods

**Figure 7:**
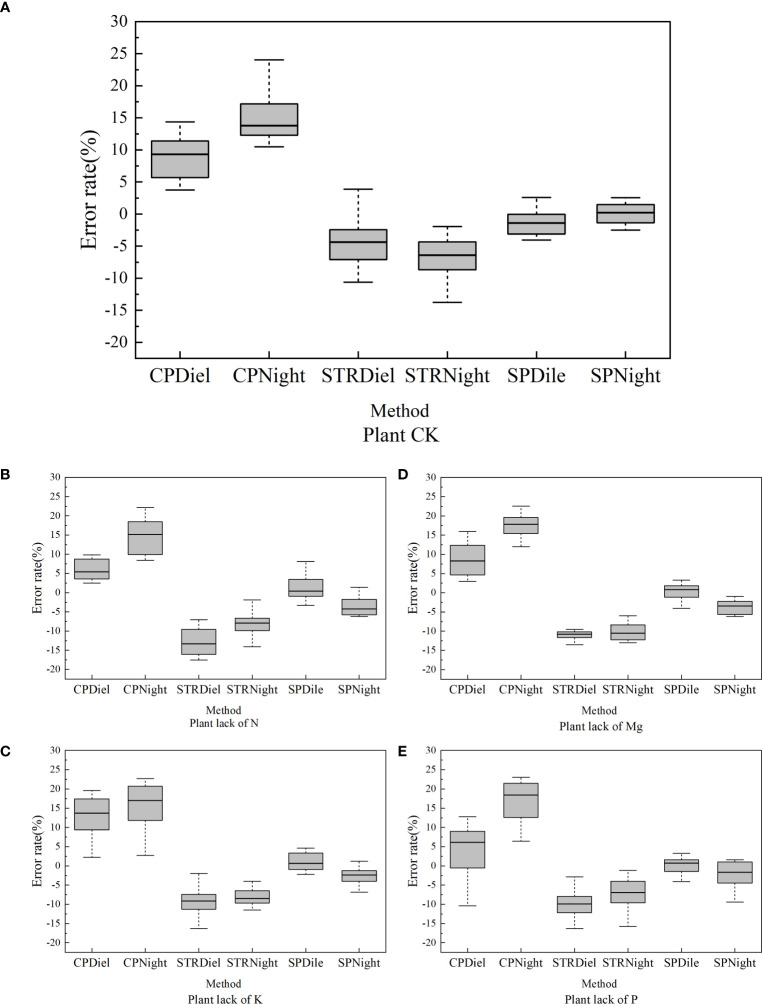
Box diagram of image segmentation error rate of rapeseed under different nutritional conditions. **(A)** Error rate under normal nutritional conditions. **(B)** Error rate under nitrogen deficiency. **(C)** error rate under potassium deficiency. **(D)** Error rate under magnesium deficiency. **(E)** Error rate under phosphorus deficiency.

The result showed that the median error rate of all three kinds of measuring methods was within 10%. The error rates of the three methods differed substantially, where a negative error rate indicates that the area measured by this method was less than the standard area, and a positive error rate indicates that the area measured by this method was greater than the standard value. The results showed that the stretching method had a negative error for leaf area measurement. The measured leaf area data were smaller than the area data obtained from the analysis with the leaf area scanning software. As shown in **Figure 7A**, in the “ck” group, during the day, the average error of the manual method was 9.13%, the average error of the stretching method was 4.60%, and the average error of the splint method measurement was 1.25%. The same situation can be seen at night. During the night, the average error of the manual method was 15.10%, the average error of the stretching method was 6.66%, and the average error of the splint method measurement was 0.05%. In other words, the average error rate of the manual method was 12.12%, the average error rate of the stretching method was 5.63%, and the average error rate of the splint method was 0.65%.

The error rate of the manual method was positive in all five treatments. Another interesting aspect of this graph was that, when using the stretching method, the predicted area was always lower than the area predicted by the scanning method. Therefore, when images of leaves were obtained, the clips would cover part of the leaf, and during the image process, this part of the leaf would not be classified to be the leaf part. Then, the area of the leaf predicted by the stretching method was lower than the area measured by the scanner. What is striking in this figure is that the error rate of the SP method was ±%5, which is much better than that of either of the other two methods.

Another point that needed to be considered was that the error rate varied with the treatment that the plants were taken in, and the explanation for this phenomenon is that the leaves’ shape and size vary when their images are captured. This is particularly true when they are been under nutrient stress such as a lack of nitrogen, phosphorus, potassium, and magnesium. The young plant of rapeseed shows different symptoms when deficient in nutrients, especially on the leaves. When they lack nitrogen, the leaves are yellow; when they lack potassium, the marginal part of the leaves is yellow; when they lack phosphorus, the leaves are curled; and when they lack magnesium, the leaves are partly yellow. Therefore, leaf deficiency symptoms have some influence on the prediction of leaf area, and there is a normal phenomenon in which the error rate is different when the treatment varies.

According to the results mentioned above, the area measured by the SP method is closer to the real area of the leaf.

### 3.2 Model training and validation

To make a comparison with the U-Net-attention algorithm proposed in this study, we also used deep learning segmentation methods such as PSPNet to process the images. By comparing the model segmentation indices, we finally obtained the segmentation method with higher accuracy. According to **Table 2**, we can see that the proposed U-Net-attention semantic segmentation model with a fused attention mechanism had more advantages.

**Table 2:** Segmentation results on different models.

Model	mIoU	mPA	mPrecision	Recall	Time (s)
HRnet	82.37%	84.31%	94.45%	68.78%	1.09
DeepLab v3+	88.50%	90.21%	95.99%	80.55%	0.76
PSPNet	75.88%	77.42%	95.70%	54.93%	1.08
U-Net	98.09%	99.32%	97.53%	98.74%	1.39
U-Net-Attention	98.58%	99.42%	98.34%	98.90%	1.37

**Figure 8:**
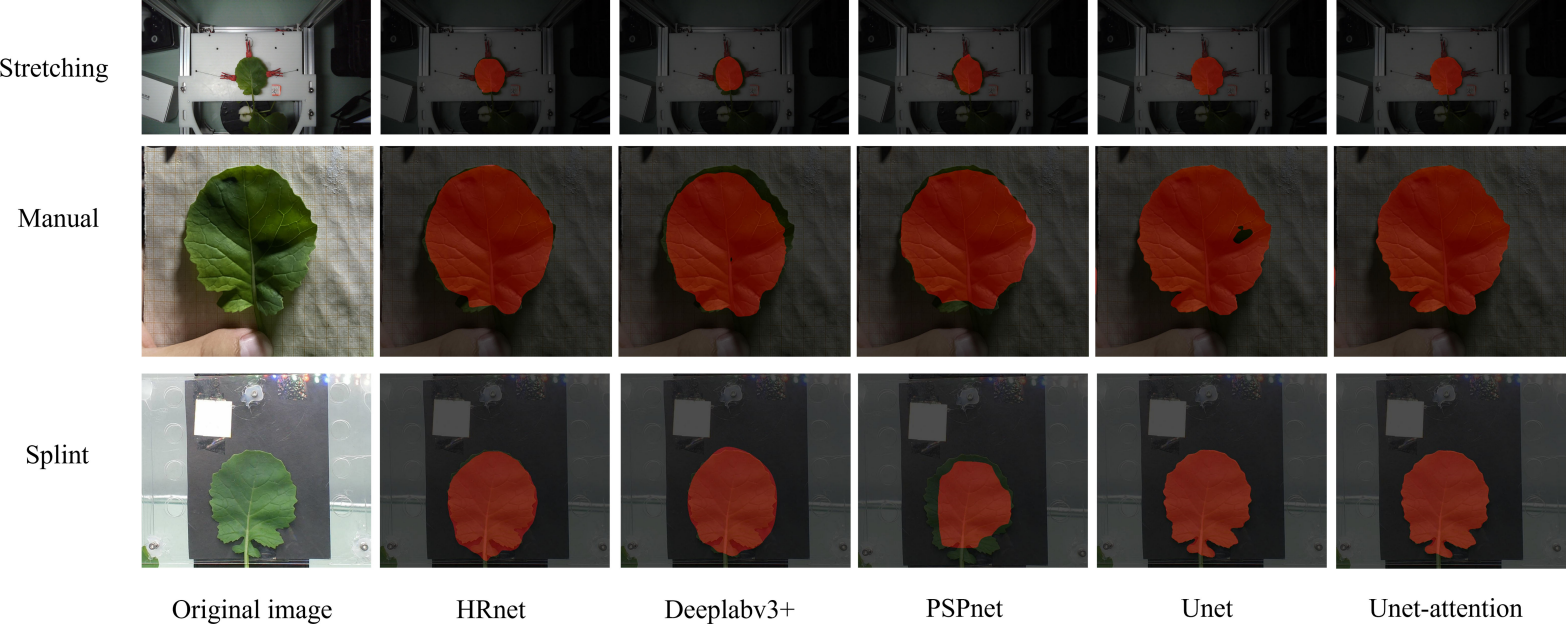
Image segmentation results.

The segmentation result is shown in **Figure 8**. The semantic segmentation model based on the U-Net-attention model well-segmented the leaf area in the image, and the application effect in the splint method was suitable, which could meet the requirement of calculating the area accuracy. HRnet could segment the leaf region roughly, but there was some loss of leaf edge detail. DeepLab v3+ had a slightly worse segmentation effect than HRnet, and the degree of edge loss was more serious. PSPNet has a better segmentation effect in the stretching and manual segmentation.

The models used in the above three segmentation models did not perform well in the dataset of this experiment. The U-Net model performed well on the dataset with a small sample size. As shown in **Figure 7**, the model segmentation effect of the U-Net and U-Net-attention model was significantly better than the other three models, but the U-Net model without the attention mechanism appears less vacant for rapeseed segmentation, and the addition of the attention mechanism eliminates this phenomenon.

We also tested the time to process the images for each prediction model. There is little difference in the running time of U-Net attention with the addition of the attention mechanism compared to that without it. Although HRnet, DeepLab v3+, and PSPNet have improved versions in prediction time due to U-Net and its addition of the attention mechanism since the current study focuses on leaf segmentation, the segmentation accuracy is used as the main evaluation index, and U-Net-attention model is used as the optimal segmentation model.

### 3.3 Effects of different measuring methods on growth

The purpose of this experiment was to compare the effect on the growth rate of plants with different measuring methods on oilseed rapeseed. We started monitoring the leaf area on the first day as a standard and compared the change in leaf area for the following 7 days.

**Figure 9:**
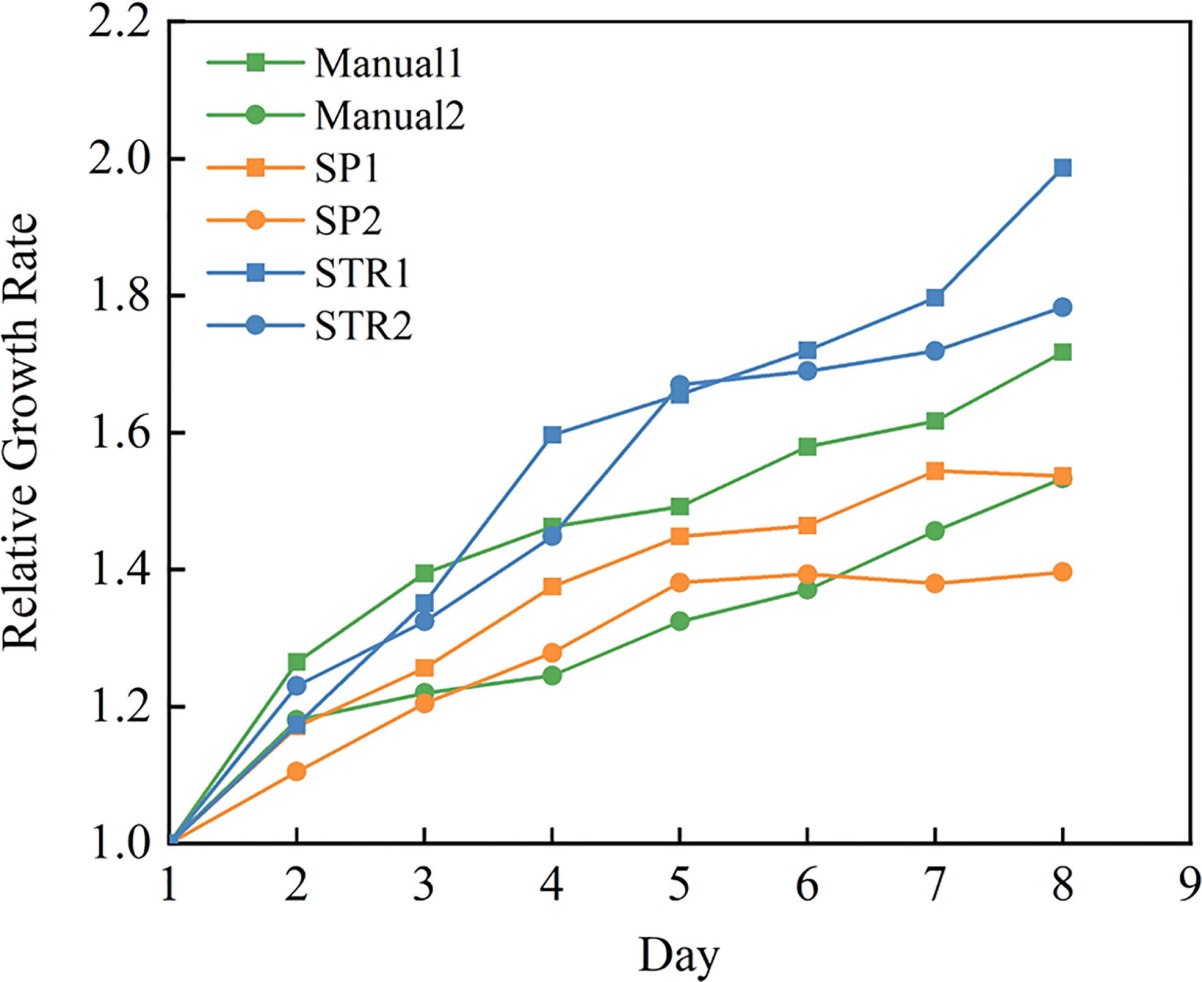
Growth rate of the three measuring methods.

The growth rate of rapeseed was measured under three methods, with two replicates of each method, and the middle value of three repetitions was taken for analysis. The leaf area of each leaf was measured by various measuring methods at 16:00 every day. **Figure 9** provides an overview of the effects of three measuring methods on the growth rate of rapeseed. **Figure 9** reveals that there was a substantial difference between the manual method, stretching method, and splint method. As seen in the figure, there was a steady rise in all three plant treatments, which means that there was no apparent inhibition of plant growth regardless of the measuring method we used.

What is striking is that compared with the manual method, plants measured by the splint method had a lower growth rate than plants measured by the manual method and the stretching method. This may be due to the effect that the splint had on the leaf in vertical growth since the splint applies restraint in the vertical direction of the leaf to ensure that the leaf was flat. Although this had some effect on the growth of rapeseed leaf, it ensured the accuracy of leaf area measurement.

## 4 Discussion

Rapid and non-destructive measurement of leaf area is essential for monitoring plant growth rate, and growth rate monitoring provides agricultural producers with a means to monitor plant status and growth so they can more accurately plan and manage the crop production process (Karimi and Siddique, 1991).

In this study, an image processing technology-based rapeseed leaf area measurement method was proposed. The U-Net-Attention learning model with an attention block was used for an initial segmentation of the original image; then, an image processing algorithm was used to perform binarization and hole-filling operations on the image; finally, the leaf area based on the number of leaf pixels was compared with the calibrated pixels. Based on this measurement method, leaf area measurement and continuous area change monitoring of rapeseed under different measurement conditions were completed. With the leaf area obtained from the scanner as the benchmark, the average accuracy of the leaf segmentation of the algorithm proposed in this paper was 96.77%, which was higher than the accuracy of other segmentation models. Meanwhile, rapeseed leaf monitoring experiments based on the splint method obtained more objective patterns, which laid the foundation for further leaf growth monitoring experiments based on image processing.

Other than that, the manual method (Wang et al., 2019; Lu et al., 2022) and stretching method (Ainsworth et al., 2005; Walter et al., 2008) had been used in the previous research. Manual measurement methods require cutting the leaves from the plant or using a camera to photograph the leaves in the original location; then, using professional image processing software can accurately measure the area of the cut leaves, but the absence of the leaves destroyed the natural growth state of the plant, so continuous observation in its natural state is not possible. Although the use of a camera to photograph the leaves in the original location ensures that the natural growth of the plant state maximally, the operator cannot make sure that the camera is perpendicular to the leaf and the leaf for the flat state. In **Figure 9**, we can see that the area measured by the manual method was larger than the ground true area. The stretching method could ensure the flatness of the leaf and camera when taking images, but the tension of the leaf caused a slight increase in leaf growth rate according to **Figure 9**. The splint method proposed in this paper can accomplish the measurement of rapeseed leaf area at a lower cost and reduce labor intensity. At the same time, it had less effect on leaf growth compared to the stretching method and manual method. Although the splint method had a slight effect on rapeseed leaf growth, according to the measurement accuracy, the splint method was superior to the other two measuring methods.

Many excellent methods have been proposed in the research for leaf monitoring. These include leaf area measurement by stretching the leaves (Ainsworth et al., 2005; Walter et al., 2008) and obtaining plant morphological information using 3D modeling techniques (Apelt et al., 2015; An et al., 2017; Boukhana et al., 2022). As can be seen from **Figure 9**, the stretching measurement for the leaf may destroy the original growth pattern of the leaf, while the leaf growth is a continuous process, and the rate of area change is a measure of the growth state, which requires the measurement of leaf area without affecting the original growth state of the leaf as much as possible. 3D modeling technologies can reconstruct the leaf morphology and obtain the leaf area without touching the plant, and the impact on the plant caused by its impact on the plant is almost negligible. However, the cost of expensive equipment makes it difficult to deploy this method on a large scale in the laboratory. Deep learning techniques, which have gradually become a hot research direction in recent years, can solve most of the problems that are difficult to overcome in traditional image processing methods (Baar et al., 2022; Sapoukhina et al., 2022; Tamvakis et al., 2022).

Many deep learning models had been used in the monitoring task of plants (Halstead et al., 2021; Sapoukhina et al., 2022). Some common models such as U-Net, DeppLab v3+, PSPNet, and HRnet had been widely used in leaf segmentation and disease diagnosis tasks. Deep learning model accuracy comparison in this paper showed that the U-Net model had the best performance, while PSPNet had the worst results. In addition, U-Net and DeepLab v3+ models perform well in the leaf disease segmentation task (Divyanth et al., 2023). Therefore, U-Net and DeepLab v3+ could be the preferred models for plant leaf image segmentation tasks. In this paper, the attention mechanism was incorporated into the U-Net model to form the U-Net-Attention model, and it can be seen from **Table 2** that the U-Net-Attention model had higher accuracy. Incorporating attention mechanisms into models to improve segmentation accuracy is a more common approach in deep learning, and in order to adapt to specific segmentation tasks, Mishra added the attention mechanism into the original model, which further improves the segmentation accuracy (Mishra et al., 2021). In future work, we can try to add the attention module to different positions in the model to obtain better segmentation effects.

**Figure 9** shows that although the splinting method proposed in this paper can increase the measurement accuracy of rapeseed leaf to an extent, there is still a certain inhibition effect of splinting on leaf growth compared to the control group. In future work, we need to further explore better ways to fix the leaves and make non-destructive measurements with minimum effect on leaf growth.

The use of non-contact methods to obtain plant phenotype information has always been a concern for scholars, and non-contact measurement methods are a key aspect of the non-destructive measurement of plant phenotypes. With the development of image processing technology, an increasing number of high-throughput computer vision-based plant monitoring devices are appearing in the agricultural field, which enables researchers to reach a new level of research on plant growth process monitoring(Zhang et al., 2021).

However, the most advanced methods currently focus on a two-dimensional analysis and three-dimensional model building of plant structure and morphology. There are fewer studies on the growth monitoring of individual plant organs. The leaves, stalks, flowers, and fruits of plants show different behaviors based on environmental stresses, which affect the physiological and biochemical processes of the whole plant and influence the growth of the plant. The splint method performed in this paper provided a good way to monitor the phenotypic characteristics of plants. Therefore, in the future, this method could be used in plant disease monitoring.

## Data availability statement

The raw data supporting the conclusions of this article will be made available by the authors, without undue reservation.

## Author contributions

ML, YL, and ZL conceived the idea, proposed the method, and revised the paper. MS and HL contributed to the preparation of the experiment and the acquisition of data. ML wrote the paper. All authors contributed to the article and approved the submitted version.
